# Nanocomposite of Half-Fin Anchovy Hydrolysates/Zinc Oxide Nanoparticles Exhibits Actual Non-Toxicity and Regulates Intestinal Microbiota, Short-Chain Fatty Acids Production and Oxidative Status in Mice

**DOI:** 10.3390/md16010023

**Published:** 2018-01-11

**Authors:** Ru Song, Jianbin Yao, Qingqing Shi, Rongbian Wei

**Affiliations:** 1 Key Laboratory of Health Risk Factors for Seafood of Zhejiang Province, College of Food Science and Pharmacy, Zhejiang Ocean University, Zhoushan 316000, China; 18368082501@163.com (J.Y.); sqq930302@163.com (Q.S.); 2 College of Marine Science and Technology, Zhejiang Ocean University, Zhoushan 316000, China

**Keywords:** half-fin anchovy hydrolysates, zinc oxide nanoparticles, acute toxicity, 16S rRNA gene sequencing, short chain fatty acids, oxidative status

## Abstract

The nanocomposite of half-fin anchovy hydrolysates (HAHp) and zinc oxide nanoparticles (ZnO NPs) (named as HAHp(3.0)/ZnO NPs) demonstrated increased antibacterial activity compared to either HAHp(3.0) or ZnO NPs as per our previous studies. Also, reactive oxygen species (ROS) formation was detected in *Escherichia coli* cells after treatment with HAHp(3.0)/ZnO NPs. The aim of the present study was to evaluate the acute toxicity of this nanocomposite and to investigate its effect on intestinal microbiota composition, short-chain fatty acids (SCFAs) production, and oxidative status in healthy mice. The limit test studies show that this nanoparticle is non-toxic at the doses tested. The administration of HAHp(3.0)/ZnO NPs, daily dose of 1.0 g/kg body weight for 14 days, increased the number of goblet cells in jejunum. High-throughput 16S ribosomal RNA gene sequencing of fecal samples revealed that HAHp(3.0)/ZnO NPs increased Firmicutes and reduced Bacteriodetes abundances in female mice. Furthermore, the microbiota for probiotic-type bacteria, including *Lactobacillus* and *Bifidobacterium*, and SCFAs-producing bacteria in the Clostridia class, e.g., *Lachnospiraceae_unclassified* and *Lachnospiraceae_UCG-001*, were enriched in the feces of female mice. Increases of SCFAs, especially statistically increased propionic and butyric acids, indicated the up-regulated anti-inflammatory activity of HAHp(3.0)/ZnO NPs. Additionally, some positive responses in liver, like markedly increased glutathione and decreased malonaldehyde contents, indicated the improved oxidative status. Therefore, our results suggest that HAHp(3.0)/ZnO NPs could have potential applications as a safe regulator of intestinal microbiota or also can be used as an antioxidant used in food products.

## 1. Introduction

ZnO, generally regarded as a safe (GRAS) material by the United States Food and Drug Administration (USFDA, 21CFR182.8991), is the most widely used nanoparticle [1]. The antimicrobial property of ZnO nanoparticles (NPs) makes them a viable approach to prevent infectious diseases [2]. To date, ZnO NPs have been developed as functional nanofillers that are incorporated into packaging and food contact materials to inhibit target bacteria [3,4]. However, it is also known that NPs might pose deleterious effects to living beings. In a study by Wang et al. (2006), the gastrointestinal (GI) administration of nanoscale zinc (N-Zn) powder on healthy adult mice for two weeks resulted in the deaths of two mice after the first week of treatment. Furthermore, N-Zn powder administration caused pathological lesions in the liver, renal, and heart tissues [5].

When NPs are in protein or peptide solutions, they could be surrounded and modified with a biocompatible ligand or a polymer to establish a dynamic layer on the surface of NPs, forming what is known as the protein “corona” [6,7]. The formation of the “corona” on the surface of the NPs might not only reduce the toxicity but also increase the biological activity of NPs. For example, Fu et al. (2016) described RGD-peptide-functionalized SeNPs as carriers of anticancer drugs that enhanced cellular uptake and antiangiogenic activities and effectively reduced toxicity in vivo [8]. The modification of bovine serum albumin on ZnO NPs significantly decreased their cytotoxicity [9]. The large specific surface area and high surface energy of ZnO NPs can contribute to their excellent interfacial interactions on polymer branches [10]. In our previous studies, we reported the nanocomposite of half-fin anchovy hydrolysates (HAHp) with ZnO NPs via a simple and green hydrothermal method, and the generated novel nanocomposite demonstrated increased antibacterial activity compared with HAHp or ZnO NPs alone [11].

Intestinal microbiota, a highly active society of organisms with a total of 10^14^ cells colonizing the gastrointestinal (GI) tract, possesses a mutualistic relationship with the host [12,13]. The intestinal microbiota composition of humans or animals can be altered through dietary or food supplementation [14]. In recent years, intestinal microbiota has gained great attention due to its impact on host metabolism, physiology, and immune system development [15,16,17]. Alterations in the composition and function of intestinal microbiota have been linked to malignancies and disorders, such as obesity and diabetes [18,19], neurodevelopmental disorders [20], cirrhosis and chronic liver diseases [21], cardiovascular diseases [22] and GI cancers [23]. It is estimated that less than 30% of gut microbial populations have been successfully cultured in vitro because most gut microorganisms are strictly anaerobic bacteria and therefore difficult to culture unless they are developed in optimal growth conditions [24]. Recently, sequencing-based molecular technologies have been created and successfully applied to the analysis of the intestine’s complex bacterial ecosystem to illustrate how the interaction between microbiota and food affects host physiology, and thus provides useful information for the potential prevention of multiple diseases [25]. A number of studies have reported that the changes of four major phyla (Actinobacteria, Bacteroidetes, Firmicutes, and Proteobacteria) are associated with physiology disorders and diseases [26,27]. Furthermore, the abundance of specific bacteria species is related to the corresponding positive or negative effects in individuals. For example, the genus *Faecalibacterium* is characterized by anti-inflammatory activity within the gut [28]. The Lachnospiraceae family is well known to participate in the breakdown of carbohydrates into short-chain fatty acids (SCFAs) [29]. A decrease in Bacteroides is suggested to be associated with metabolic diseases, such as obesity and diabetes [30,31].

HAHp, the digestion products of half-fin anchovy proteins hydrolyzed by pepsin, are composed of peptides and amino acids, and they have demonstrated antibacterial, antioxidant, and anti-proliferative activities [32,33,34]. Although the antibacterial activity of HAHp was increased after conjugating with ZnO NPs [11], to our knowledge, there are few studies about the acute toxicity of the conjugates of ZnO NPs with peptides or proteins, as well as analyses of intestinal microbiota composition after short-term continuous administration of peptide or protein/ZnO NPs. Therefore, the aim of this study was to assess the acute toxicity of the nanocomposite of HAHp and ZnO NPs (named as HAHp(3.0)/ZnO NPs). Furthermore, the high-throughput 16S ribosomal RNA (rRNA) gene sequencing technique was applied to investigate the presence of specific alterations in the intestinal microbiota after the administration of this nanocomposite. Moreover, the change of liver oxidative status was also determined to reveal a possible association with intestinal microbiota alterations.

## 2. Results

### 2.1. Acute Oral Toxicity of HAHp(3.0)/ZnO NPs

Prior to the acute toxicity study, the hemolytic rate of HAHp(3.0)/ZnO NPs in the red blood cells of the mice was determined. At the concentration of 1.0 g/mL, no hemolysis was observed. In the acute toxicity study, we used the limit test to evaluate the acute toxicity of HAHp(3.0)/ZnO NPs in mice. HAHp(3.0)/ZnO NPs were dissolved in saline and administrated via gavage to reach a dose of 10.0 g/kg body weight (BW) (zinc content of 912.74 mg/kg BW).

During 14 days of administration, no treatment-related clinical signs of toxicity or mortality were observed. In addition, no weight loss was detected in the same sex of mice, although male mice had higher body weights than female mice (*p* < 0.05) (Table 1). Similarly, no significant differences were found for the liver coefficient and the thymus coefficient in the female and male mice between the normal control (CK) and HAHp(3.0)/ZnO NPs groups. Compared with the female mice, a decrease of the spleen coefficient in the male mice in both the CK and HAHp(3.0)/ZnO NPs groups partially contributed to their simultaneous increase in body weight. However, no significant differences were observed within the mice of the same sex (*p* > 0.05). According to the standard in the Procedure and Methods of Food Safety Toxicological Assessment, GB15193.3-2014 (in Chinese), the median lethal dose (LD_50_) > 5000 mg/kg BW belongs to the actual non-toxic level. In the present study, based on the results of the limit test, the LD_50_ of HAHp(3.0)/ZnO NPs in mice was above 10.0 g/kg BW, suggesting its safety.

### 2.2. Histopathology

**Figure 1:**
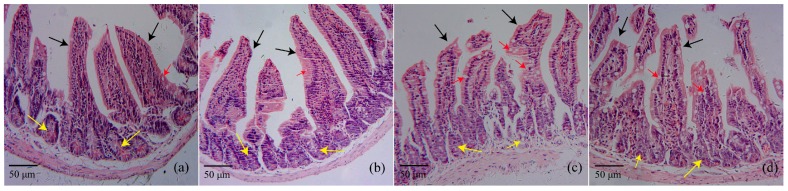
Histopathology of mice jejunum showing intestinal villi (black arrow), crypt regions (yellow arrow), and goblet cells (red arrow) from (**a**) female normal control, CK(F); (**b**) male normal control, CK(M); (**c**) HAHp(3.0)/ZnO NPs administered female mice; and (**d**) HAHp(3.0)/ZnO NPs administered male mice. HE stain at the initial magnification 200×.

After continuous oral administration of HAHp(3.0)/ZnO NPs for 14 days, the morphological changes of the jejunum were observed with hematoxylin-eosin (HE) stain (Figure 1). In the female normal control CK(F) and male normal control CK(M) groups, typical microvilli lined by enterocytes bearing brush border membranes and intestinal crypt regions having different types of cells can be seen in Figure 1a,b. The gavage administration of HAHp(3.0)/ZnO NPs in mice for 14 days at a dose of 1.0 g/kg BW did not lead to marked degeneration and desquamation of the intestinal villi and crypt regions of the jejunum. However, a profoundly increased number of goblet cells were observed in microvilli lines after HAHp(3.0)/ZnO NPs administration (Figure 1c,d).

### 2.3. Effect of HAHp(3.0)/ZnO NPs on the Alteration of Intestinal Microbiota Composition

#### 2.3.1. Species Abundance and Diversity

**Figure 2:**
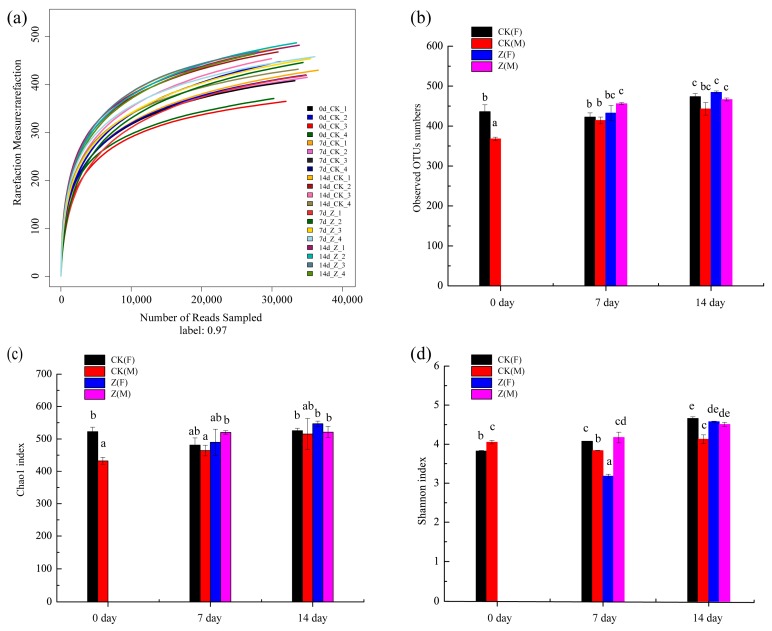
Rarefaction curves and diversity of the normal control (CK) and HAHp(3.0)/ZnO NPs treated (Z) specimens at 0 day, 7 day, and 14 day. (**a**) Rarefaction curves, numbers 1 and 2 represent female (F) specimens and numbers 3 and 4 represent male (M) specimens. Horizontal coordinate represents the number of sequencing reads randomly sampled and the longitudinal coordinate means the number of operational taxonomic units (OTUs) observed; (**b**) comparison of observed OTUs numbers among CK and Z specimens based on sex; (**c**) community richness based on the Chao1 index; (**d**) community diversity based on the Shannon index. Different lowercase letters in (**b**–**d**) indicate significant differences among specimens (*p* < 0.05).

A total of 649,051 valid sequences were determined for the 20 specimens. The average number of sequences of each specimen was more than 30,000 sequences. Of the sequence detected, 100% were >400 bp in length. The plateau phase of rarefaction curve suggests the adequate sequencing reads in the experiment [35]. Obviously, most specimens in the rarefaction curves reached the plateau phases (Figure 2a), confirming the adequate sequencing depth of the experiment. The observed OUTs numbers between the CK and the HAHp(3.0)/ZnO NPs treated (Z) specimens were further compared, shown in Figure 2b. Significant differences were observed in CK(F) and CK(M) at 0 day. At the seventh and 14th days, the observed OTUs in all of the Z specimens were higher than the same sex specimens in the CK groups.

Similar to the results shown in Figure 2b, the CK groups showed significant differences in the Chao1 indices between the female and male specimens at 0 day (*p* < 0.05), and consistent tendencies with the results shown in Figure 2b were observed at 7 day and 14 day (Figure 2c). A greater Shannon index indicates a greater diversity of intestinal microbiota [36]. The Shannon indices of all of the specimens changed with the feed time. The Shannon index of Z(M) at 7 day was 4.17 ± 0.14 and it increased to 4.51 ± 0.05 at 14 day. In comparison, the Shannon index of Z(F) was dramatically increased from 3.18 ± 0.05 at 7 day to 4.58 ± 0.02 at 14 day (*p* < 0.05) (Figure 2d).

#### 2.3.2. Principal Coordinates Analysis (PCoA)

**Figure 3:**
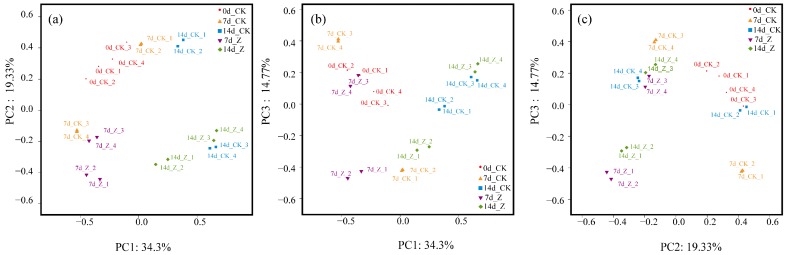
PCoA distribution of the normal control (CK) and HAHp(3.0)/ZnO NPs treated (Z) specimens at 0 day, 7 day, and 14 day. Numbers 1 and 2 represent female (F) specimens and numbers 3 and 4 represent male (M) specimens. (**a**) The scatter of PC1 and PC2; (**b**) the scatter of PC1 and PC3, and (**c**) the scatter of PC2 and PC3.

As shown in Figure 3, PC1, PC2, and PC3 accounted for 34.3%, 19.33%, and 14.77% of the variation, respectively. In the scatter of PC1 and PC2 (Figure 3a), the CK specimens at 0 day were distinguished from the CK specimens at 14 day. This indicated that the distributions of genera in the CK were changed with the feed time. Furthermore, the female specimens of HAHp(3.0)/ZnO NPs at 14 day (14d_Z_1 and 14d_Z_2) were separated from the female ones of CK (14d_CK_1 and 14d_CK_2) in the scatters of PC1 and PC2 (Figure 3a), PC1 and PC3 (Figure 3b), and PC2 and PC3 (Figure 3c). These results suggested that the administration of HAHp(3.0)/ZnO NPs could result in changes of intestinal microbiota at the genus level for female mice. By comparison, the overlap of male specimens at 14 day between CK (14d_CK_3 and 14d_CK_4) and HAHp(3.0)/ZnO NPs (14d_Z_3 and 14d_Z_4) indicated their similar intestinal microbiota composition.

#### 2.3.3. Composition of the Intestinal Microbiota at the Phylum and Genus Levels

**Figure 4:**
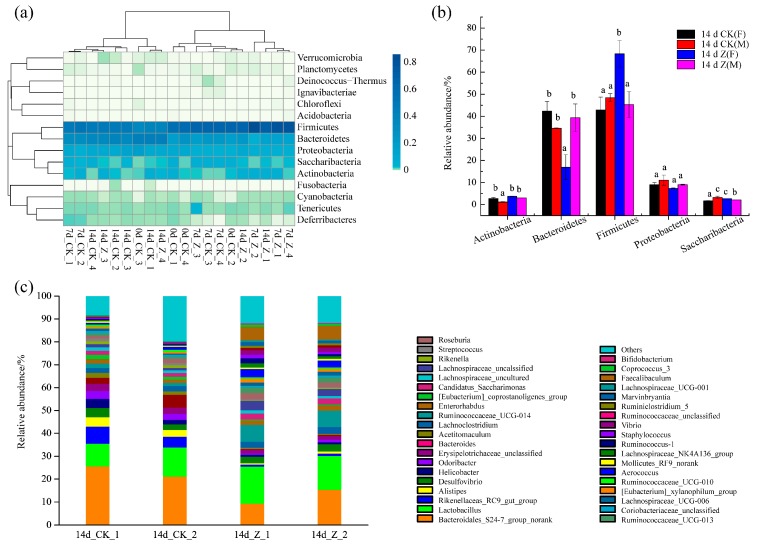
Structure and relative abundance of intestinal microbiota at the phylum and genus levels. (**a**) Intestinal microbiota composition of all of the specimens at 0 day, 7 day, and 14 day at the phylum level. Numbers 1 and 2 represent the female (F) specimens and numbers 3 and 4 represent male (M) specimens; (**b**) comparison of relative abundance of dominant bacteria at the phylum level between the normal control (CK) and HAHp(3.0)/ZnO NPs treated (Z) specimens at 14 day. The relative abundance was calculated from the abundance of 16S rRNA gene sequences assigned to each bacterial community using the Greengenes database. Different lowercase letters in each phylum bacteria indicate significant differences among specimens (*p* < 0.05); (**c**) composition of intestinal microbiota at the genus level for the female specimens of 14d_CK_1 and 14d_CK_2 and 14d_Z_1 and 14d_Z_2.

A total of 15 intestinal microflora were identified at the phylum level in the specimens (Figure 4a), of which the phyla Firmicutes, Bacteroidetes, Proteobacteria, Saccharibacteria, and Actinobacteria were most abundant, accounting for >97% of the total sequences. The other phyla had comparatively small numbers. The relative abundance of the five dominant phyla at 14 day was compared between the CK and Z groups (Figure 4b). Obviously, Firmicutes and Bacteroidetes were the two most dominant phyla in the specimens. At 14 day, the relative abundance of phylum Firmicutes were 42.89 ± 5.85% for 14 day of CK(F)(14 d CK(F)), 48.40 ± 1.83% for 14 day of CK(M) (14 d CK(M)), and 45.35 ± 5.89% for 14 day of Z(M) (14 d Z(M)). However, the relative abundance of phylum Firmicutes in 14 day of Z(F) (14 d Z(F)) was 68.32 ± 5.98%, dramatically higher than other specimens (*p* < 0.05). In contrast, a markedly decreased relative abundance of phylum Bacteroidetes (16.98 ± 5.63%) was found in 14 d Z(F) (*p* < 0.05).

The intestinal microbiota composition of 14 d CK(F) (14d_CK_1 and 14d_CK_2) and 14 d Z(F) (14d_Z_1 and 14d_Z_2) specimens was further evaluated at the genus level. As shown in Figure 4c, more than 38 bacteria at the genus level were identified in these specimens, of which *Bacteroidales_S24−7_group_norank* and *Lactobacillus* were the two most abundant bacteria communities in each specimen. The relative abundance shifts of 37 dominant bacteria between 14 d CK(F) and 14 d Z(F) at genus level plus *Bifidobacteria*, one of most important probiotic species of the intestinal tract that is associated with a healthy status in animals or humans [37], are listed in Table 2.

The microbes of groups 14 d CK(F) and 14 d Z(F) were derived from six different phyla, 10 different classes, 12 different orders, and 18 different families. Although an increase in the phylum Firmicutes was noted in 14 d Z(F) (as shown in Figure 4b), increase and decrease of particular taxa were also observed in the Bacilli, Clostridia, and Erysipolotrichi classes (Table 2).

In the Bacilli class, an increase in the relative abundance of genus *Lactobacillu* was observed in 14 d Z(F) (Table 2). Specific members including *Lachnospiraceae_unclassified*, *Lachnospiraceae_UCG-001*, *Ruminococcaceae_UCG-014*, and *Ruminococcus_1*, were markedly increased in the Clostridia class. Alternatively, some members of this class such as *Acetitomaculum* and *[Eubacterium]_coprostanoligenes_group* were reduced by the administration of HAHp(3.0)/ZnO NPs. In the Erysipelotrichia class, the relative abundance of the genus *Faecalibaculum* was as small as 0.13% in 14 d CK(F) and increased to 5.95% in 14 d Z(F).

Within the phylum Bacteroidetes, the relative abundance of all of the members of four different families, including Bacteroidaceae, Bacteroidales_S24-7_group, Porphyromonadaceae, and Rikenellaceae were declined, and significant changes were found for the genera *Bacteroides*, *Bacteroidales_S24-7_group_norank*, *Alistipes*, *Rikenella*, and *Rikenellaceae_RC9_gut_group*. In phylum Actinobacteria, the relative abundance of genera *Enterorhabdus* and *Bifidobacterium* were dramatically increased. In addition to significant shifts of some genera of the phyla Firmicutes, Bacteroidetes, and Actinobacteria, a markedly increased relative abundance of the genus *Vibrio* (phylum Proteobacteria) was observed in 14 d Z(F).

### 2.4. Function Prediction, SCFAs and Amino Acid Concentrations in Feces

**Figure 5:**
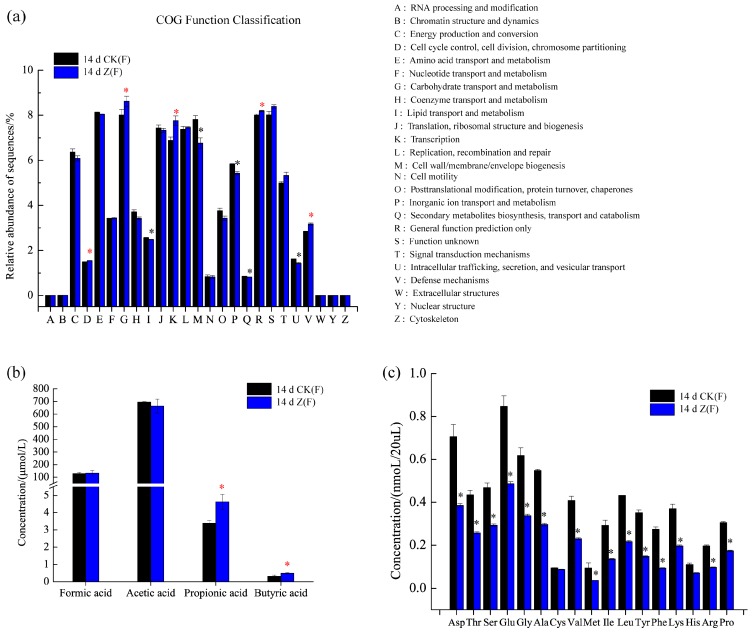
Comparison of function prediction, short-chain fatty acids (SCFAs) and amino acid concentrations in feces of female mice between 14 d CK(F) and 14 d Z(F). (**a**) Function prediction using Clusters of Orthologous Groups of proteins (COG) orthology; (**b**) SCFAs concentration; (**c**) amino acid concentrations of aspatic acid (Asp), threonine (Thr), serine (Ser), glutamic acid (Glu), glycine (Gly), alanine (Ala), cysteine (Cys), valine (Val), methionine (Met), isoleucine (Ile), leucine (Leu), tyrosine (Tyr), phenylalanine (Phe), lysine (Lys), histidine (His), arginine (Arg), and proline (Pro). An asterisk represents a significant difference between 14 d CK(F) and 14 d Z(F) in relative abundances or SCFAs, and amino acid concentrations (*p* < 0.05). The red and black asterisks in (**a**) represent significantly decreased and increased relative abundances, respectively. The red asterisks in (**b**) indicate significantly increased SCFAs concentrations. The black asterisks in (**c**) indicate significantly decreased amino acid concentrations.

Since different species of intestinal microbiota might perform same function, the function differences among individual feces can be discovered through metagenomic study. As shown in Figure 5a, the relative abundances of five categories, namely, function D (Cell cycle control, cell division, chromosome partitioning), function G (Carbohydrate transport and metabolism), function K (Transcription), function R (General function prediction only) and function V (Defense mechanisms) were markedly increased in 14 d Z(F) (*p* < 0.05). In addition to significantly increased functions, the functions of I (Lipid transport and metabolism), M (Cell wall/membrane/envelope biogenesis), P (Inorganic ion transport and metabolism) and U (Intracellular trafficking, secretion, and vesicular transport) were remarkably decreased in 14 d Z(F) (*p* < 0.05) (Figure 5a). An evident increase of SCFAs concentrations was observed in the 14 d Z(F) group, and significant increases were found in propionic and butyric acids (*p* < 0.05) (Figure 5b). As for amino acid concentration in feces, besides cysteine (Cys), other amino acids showed significantly decreased concentrations in 14 d Z(F) group compared to those in 14 d CK(F) group (Figure 5c).

### 2.5. Changes of Liver Oxidative Status

**Figure 6:**
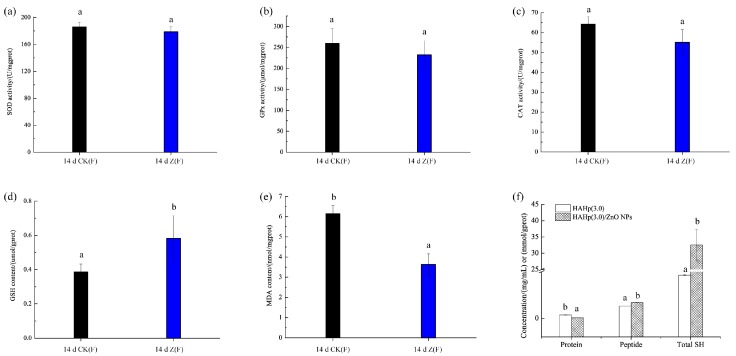
Comparison of liver oxidative status between 14 d CK(F) and 14 d Z(F) after 14 days of oral gavage at a dose of 1.0 g/kg body weight. (**a**) Total activity of superoxidase dismutase (SOD); (**b**) activity of glutathione peroxidase (GPx); (**c**) activity of catalase (CAT); (**d**) glutathione (GSH) stock content; (**e**) malonaldehyde (MDA) content. In (**a**–**e**), each group contained five animals. Different lowercase letters indicate significant differences compared between 14 d CK(F) and 14 d Z(F) (*p* < 0.05). (**f**) Concentrations of protein, peptide and total sulfhydryl (SH). Different lowercase letters in protein, peptide and total SH indicate significant differences compared between HAHp(3.0) and HAHp(3.0)/ZnO NPs (*p* < 0.05).

No significant differences were found in the total superoxide dismutase (SOD) activity (*p* > 0.05) (Figure 6a). Similar results were noted for glutathione peroxidase (GPx) and catalase (CAT) activities (Figure 6b,c), though decreased activities were observed in 14 d Z(F). However, the content of glutathione (GSH) was increased to 0.58 μmol/gprot in 14 d Z(F), markedly higher than that in the 14 d CK(F) (0.39 μmol/gprot) (*p* < 0.05) (Figure 6d). In contrast, the malonaldehyde (MDA) content of 14 d Z(F) was dramatically reduced to 3.63 nmol/mgprot, significantly lower than that in the 14 d CK(F) (6.15 nmol/mgprot) (*p* < 0.05) (Figure 6e). The total sulfhydryl (SH) content, related to GSH level and/or used as substrates for GSH biosynthesis in vivo, were further determined in HAHp(3.0)/ZnO NPs, as well as the concentrations of protein and peptide were measured (Figure 6f). Interestingly, HAHp(3.0)/ZnO NPs had significantly increased total SH content compared to HAHp(3.0) (*p* < 0.05). Meanwhile, decreased protein and increased peptide concentrations were detected in HAHp(3.0)/ZnO NPs.

## 3. Discussion

In the present study, the acute oral toxicity of HAHp(3.0)/ZnO NPs with zinc content of 912.74 mg/kg BW was consistent with a recent report by Jayarambabu, Rao and Rajendar (2018); they described that the ZnO NPs, which were synthesized by a green method using *Lawsonia inermis* plant extract, showed no mortality at 2000 mg/kg BW in an acute oral toxicity study [38]. At a dose of 1.0 g/kg BW for 14 days, a profoundly increased number of goblet cells were observed in microvilli lines. It is well known that goblet cells secrete mucins and play an important role in the innate immunological defense [39]. The positive alteration of intestinal microbiota could interfere with the production of mucus, and finally contribute to the exertion of protective effects on the integrity of the mucosal barrier function [40,41]. The increased number of goblet cells (as shown in Figure 1c,d) should be associated with the changes of intestinal microbiota composition induced by HAHp(3.0)/ZnO NPs administration. Furthermore, the results of Chao1 index and Shannon index also verified that species abundance and diversity were changed with feed time. In the PCoA analysis, the more similar two specimens are in composition in genus level, the closer they are on the PCoA diagram [36]. The result of Figure 3 revealed that the administration of HAHp(3.0)/ZnO NPs resulted in intestinal microbiota changes in mice, especially in female mice.

Dramatically increased Firmicutes and decreased Bacteroidetes were found in 14 d Z(F) compared to 14 d CK(F) or 14 d Z(M). According to some documents, in obese hosts, the relative abundance of phylum Firmicutes tends to increase, while the relative abundance of Bacteroidetes tends to decrease [42]. However, in this study, increased Firmicutes and decreased Bacteroidetes in 14 d Z(F) did not result in overweight mice (as seen in Table 1). Similar to our results, an increase in Firmicutes and a reduction in Bacteroides were observed in the immunological improvement of human fecal samples in response to the addition of whole grains to the diet [43]. In the latest study by Byerley et al. (2017), they also reported walnut consumption increased the abundance of Firmicutes and reduced the abundance of Bacteriodetes in rats, and they speculated that the reshaping of the gut microbial community may play a physiological role in promoting walnut’s health benefits [36]. The ratio of Firmicutes to Bacteroidetes (F/B) is commonly used to indicate the composition of the gut microbiota [35]. In the present study, the average F/B ratio for 14 d Z(F) was 4.32, significantly greater (>4.2-fold) than that of the corresponding 14 d CK(F) (F/B = 1.02) group. We assumed the changes of intestinal microbiota composition, especially for the phyla Firmicutes and Bacteroidetes, could be responsible for the distinctive PCoA plot profiles between 14 d CK(F) and 14 d Z(F) (as shown in Figure 3).

Although an increase in the phylum Firmicutes was noted in 14 d Z(F) (as shown in Figure 4b), particular taxa increase and decrease were also observed within the phylum Firmicutes. Significant changes were observed in the Bacilli, Clostridia, and Erysipolotrichi classes. In the Bacilli class, the Lactobacillaceae family is known to participate in the breakdown of carbohydrates into SCFAs [29]. The positive functions of various strains of lactic acid bacteria (LAB) derived from the Lactobacillaceae family have attracted great attention in the last few years. Li et al. (2017) reported that two species of *Lactobacillus*, *Lactobacillus* G15 and Q14, which are used in the preparation of Chinese traditional fermented dairy foods, alleviated type 2 diabetes in a gut microbiota-dependent way *via* down regulating G^−^ bacteria-related lipopolysaccharide secretion and promoted the enrichment of SCFA-producing bacteria [44]. Yu and Li (2016) gathered the studies of lactic acid production of LAB that could contribute to the prevention of colon cancer and modulate host immunity, anti-inflammatory, and antipathogenic activity [45]. Similar to our results, Yu, Amorim, Marques, Calhau and Pintado (2016) found the relative abundance of *Lactobacillus* spp. was significantly increased by whey peptide extract in rats fed with a standard diet [46]. In the present study, a significant increase in the relative abundance of genus *Lactobacillus* in 14 d Z(F) (as seen in Table 2) suggested potential benefits could be expected by consuming enriched HAHp(3.0)/ZnO NPs.

The proportions of bacteria in the Clostridia class were 16.48% for 14 d CK(F) and 32.19% for 14 d Z(F) based on their relative abundances in Table 2. The Clostridia class is known for its production of butyrate [36], which has multiple functions as a cellular mediator, including gut cell regulation, gut tissue development, cell differentiation, immune modulation, oxidative stress reduction, inactivation of carcinogens, as well as acting as a primary nutrient to provide energy for colonocytes [47]. The results shown in Table 2 revealed that the administration of HAHp(3.0)/ZnO NPs contributed to a higher amount of bacterial counts in the Clostridia class, which could result in an increased production of butyrate and promote positive effects on mice. Similarly, in the Erysipelotrichia class, the relative abundance of the genus *Faecalibaculum* was 0.13% in 14 d CK(F) in contrast with an increase to 5.95% in 14 d Z(F). *Faecalibacterium* was characterized by the anti-inflammatory activity within the gut [28]. Therefore, an increase in this genus indicated an elevated anti-inflammatory activity induced by the administration of HAHp(3.0)/ZnO NPs.

Although a reduction in *Bacteroides* abundance is assumed to be associated with some metabolic diseases, such as obesity and diabetes [30,31], the enriched microbiota for probiotic-type bacteria, including *Lactobacillus*, *Ruminococcaceae*, and *Roseburia*, in rat feces was induced by intake of walnuts, while it was significantly reduced in *Bacteroides* and *Anaerotruncus* [36]. Similarly, in the present study, the increased relative abundances of *Lactobacillus*, *Ruminococcaceae*, and *Roseburia* were found in 14 d Z(F), as well as a reduced relative abundance of *Bacteroides* (Table 2). Furthermore, a decrease in *Alistipes* is considered to reduce the frequency of abdominal pain in patients with irritable bowel syndrome, and thus decrease gut inflammation [48]. The decrease in the relative abundance of *Alistipes* in 14 d Z(F) also suggested its contribution to the anti-inflammatory function.

In phylum Actinobacteria, the relative abundance of genera *Enterorhabdus* and *Bifidobacterium* were dramatically increased. In particular, the genus *Bifidobacterium* had as high as 53-fold relative abundance in 14 d Z(F) after administration of HAHp(3.0)/ZnO NPs. The genus *Bifidobacterium* can secrete lacto-N-biosidase to digest oligosaccharides, such as galactooligosaccharide, to produce SCFAs [49,50]. A number of studies have proven the beneficial effects of the *Bifidobacterium* species on the immune system and allergic diseases later in life [51,52]. However, the number of *Bifidobacterium* dominant in an infant’s gut microbiota, decreased with solid food consumption and old age in animals and humans [53]. The enriched genus *Bifidobacterium* in 14 d Z(F) gut microbiota should be attributed to the prebiotic effect induced by HAHp(3.0)/ZnO NPs administration.

In addition to significant shifts of some genera of the phyla Firmicutes, Bacteroidetes, and Actinobacteria, a markedly increased relative abundance of the genus *Vibrio* (phylum Proteobacteria) was observed in 14 d Z(F). In the genus *Vibrio*, there are over 100 individual species, and only a few of them have been associated with human illness [54]. In fact, specific bacteria of *Vibrio* demonstrated positive effects, for instance, Liu, Zhang, Zhang, and Li (2017) recently reported the bacterium *Vibrio* sp. V33 isolated from healthy sepia showed strong antagonistic activity towards the pathogenic isolate *Vibrio splendidus* [55]. During the oral administration of HAHp(3.0)/ZnO NPs in mice, we did not observe treatment-related clinical signs of toxicity or mortality in 14 d Z(F). We speculated that the increase in genus *Vibrio* might play weak roles in the health of the mice.

In the present study, the potential microbial functions of 14 d CK(F) and 14 d Z(F) were identified by Phylogenetic Investigation of Communities by Reconstruction of Unobserved States (PICRUst) 1.1.3 documentation [56] (as shown in Figure 5a). A total of 25 function categories were predicted in both 14 d CK(F) and 14 d Z(F) fecal samples. It was noteworthy that the increased function V in 14 d Z(F) suggested the contribution of HAHp(3.0)/ZnO NPs administration in the promotion of individual health. This result was consisted with the increased number of goblet cells (as shown in Figure 1c), which could facilitate the innate immunological defense. Furthermore, the increase of predicted function G (Carbohydrate transport and metabolism) (Figure 5a) and the decrease of detected amino acids in 14 d Z(F) (Figure 5c) suggested more amount of SCFAs could be produced after HAHp(3.0)/ZnO NPs administration, since SCFAs are major derived from carbohydrate fermentation and degradation of proteins by the intestinal microbiota [36]. As we expected, an evident increase in the SCFAs concentrations was observed in the 14 d Z(F) group (Figure 5b). Production of SCFAs not only can be used as energy source, but also can decrease the intestinal pH values. Different scientific publications have demonstrated that increases of SCFAs content are associated with the enhanced anti-inflammatory activity in host [57,58]. Especially, the SCFAs of acetic, propionic and butyric acids have deep impacts on the physiology and nutrition of the gastrointestinal tract [59]. Though no statistical significances were found in formic and acetic concentrations between 14 d CK(F) and 14 d Z(F) groups, significant increases of propionic and butyric acids were detected in 14 d Z(F) group (as shown in Figure 5b). Propionic and butyric acids can act as attracting molecules for neutrophils by means of the interaction with the anti-inflammatory receptor, and subsequently inhibit the inflammatory response caused by diverse agents [57,60]. The increases of propionic and butyric acids in 14 d Z(F) group meant positive effects of HAHp(3.0)/ZnO NPs in regulating intestinal microbiota composition. Also, the result of SCFAs was consistent with the upregulated genera shifts in 14 d Z(F) group (as seen in Table 2).

Among these reduced amino acids (as shown in Figure 5c), the most decreased amino acid was Phe, with concentration ranging from 13.70 nmol/mL to 4.72 nmol/mL (−65.55%). This result was consisted with the fact that phenyl-containing amino acids are a priority to be metabolized by intestinal bacteria [61]. In contrast to those significantly decreased amino acids, the concentration of Cys in 14 d Z(F) remained stable. This meant the alteration of intestinal microbiota composition induced by HAHp(3.0)/ZnO NPs administration had less effect on Cys concentration in feces.

It is well known that reactive oxygen species (ROS) generation is a predominant contributor to the antibacterial activity of ZnO NPs in vitro. In our previous studies, we proved that the incubation of HAHp(3.0)/ZnO NPs with *E. coli* cells induced ROS formation in intracellular cells [11]. Excess ROS in vivo might cause oxidative stress which is associated with the development of oxidative damage and chronic inflammation [62]. In this study, the increased propionic and butyric acids detected in the fecal samples in HAHp(3.0)/ZnO NPs fed group (as shown in Figure 5b) suggested that HAHp(3.0)/ZnO NPs could be used as a diet supplement to improve immunity and gut motility in mice. Liver is an important organ for detoxification and metabolism in animals and humans, the oxidative stress of which might be influenced by intestinal microbiota composition changes.

In the mechanism of ROS scavenging, of the key enzymes involved, including SOD, GPx, and CAT, play crucial roles in converting ROS into H_2_O and O_2_ to avoid ROS accumulation [63,64]. In the present study, the content of GSH, the most abundant low molecular weight thiol groups that protect the cells from oxidative stress damage [65], were significantly increased in 14 d Z(F) samples. Meanwhile, the MDA content, reflecting the peroxidation of polyunsaturated fatty acid in biological membrane, was decreased dramatically. In general, the MDA level is increased with aggravated oxidative stress [63]. The decreased MDA content as shown in Figure 6e indicated an increase of antioxidative status in mice liver after HAHp(3.0)/ZnO NPs administration for 14 days. Similar to our results, Tang et al. (2014) reported the supplementation with zinc-bearing clinoptilolite (ZnCP) in a basal diet for broiler chickens not only modulated microbial populations in fecal content, but also decreased the MDA content and increased the GSH content in the intestine [41]. A balance of intestinal microbiota in animals can be found to serve for various health benefits, such as modulation of redox status in the host, breakdown and synthesis of nutrients, detoxification of toxic products, prevention of infection by pathogenic organisms, and development of the host immune system [66,67,68]. Therefore, the increased populations of beneficial bacteria, such as *Lactobacillus*, *Faecalibaculum*, and *Bifidobacterium*, and the decreased numbers of harmful bacteria including *Bacteroides* and *Alistipes* in the treatment group of 14 d Z(F) (seen in Table 2) could play important roles in preventing hepatic GSH depletion, and consequently inhibited lipid peroxidation.

In addition, some specific peptides were able to elevate cellular GSH levels. For example, Shi et al. (2014) reported eggshell membrane peptides, digested with Alcalase and Protease S (AL-PS), and its ultrafiltered fraction AL-PS-I (Molecular weight cut off < 5 kDa) significantly suppressed the formation of H_2_O_2_-induced MDA and protein carbonyl derivatives, and increased GSH synthesis and antioxidant enzyme activity in H_2_O_2_-stimulated Caco-2 cells [69]. In our previous studies, we found the HAHp(3.0)/ZnO NPs displayed different transmission electron microscopy images compared to their initial counterparts of ZnO NPs, and a dynamic layer of “corona” formed on the surface of ZnO NPs [11]. Proteins and/or peptides with specific residues and conformations could be responsible for binding with ZnO NPs [70]. We speculated that specific groups in HAHp(3.0)/ZnO NPs may be another contributor to biosynthesis or accumulation of GSH in liver. Interestingly, according to the result from Figure 6f, HAHp(3.0)/ZnO NPs had significantly increased SH content compared to HAHp(3.0) (*p* < 0.05). Meanwhile, decreased protein concentrations and increased peptide concentrations were detected in HAHp(3.0)/ZnO NPs. Accordingly, we supposed that the dynamic “corona” absorbed onto the surface of ZnO NPs in HAHp(3.0)/ZnO NPs should contain large amounts of SH residues, such as Cys, GSH and/or GSH alike peptides. Since the GSH levels are highly regulated and limited by availability of the sulfhydryl amino acid l-Cys [71], the present result as seen from Figure 6f also suggested that HAHp(3.0)/ZnO NPs should be a good source of glutathione or its precursor for glutathione biosynthesis. High level of glutathione in diet is well known to act as excellent antioxidants to provide beneficial effects in vivo [72]. The relatively stable concentration of Cys in the fecal sample of 14 d Z(F) (as seen in Figure 5c) might be due to the higher SH concentration of HAHp(3.0)/ZnO NPs fed in mice. However, further studies need to investigate the adsorption kinetics of HAHp(3.0)/ZnO NPs in the intestinal tract in large animal models.

## 4. Materials and Methods

### 4.1. Materials

Half-fin anchovy (*Setipinna taty*) were purchased from Fengmao Aquatic Market in Zhoushan City, China. The extraction kits of bacterial genome DNA were bought from Beyotime Institute of Biotechnology (Haimen, China). The total SOD, GPx, CAT, GSH, MDA, and total sulfhydryl assay kits were purchased from Jiancheng Bioengineering Institute (Nanjing, China). All of the other chemicals were obtained from commercial products. Female and male Kunming species mice (clean), approximate weight 18–22 g, were purchased from the Laboratory Animal Center of Zhejiang Province, Hangzhou, China (SCXK (Zhe)2014-0001, certification number: 1612220002).

### 4.2. Preparation of the Nanocomposite of HAHp and ZnO NPs

The nanocomposite of HAHp and ZnO NPs were prepared according to our previous studies [11,32]. In brief, the pH of the HAHp was adjusted to 3.0 and centrifuged at 5000× *g* for 10 min. The supernatant was collected and designated as HAHp(3.0). The ZnO NPs were added to HAHp(3.0) to reach the final concentration of 19.78 mg/mL. After 6.9 h of incubation at 37 °C in a water bath oscillator (160 rpm), the mixtures were centrifuged at 5000× *g* for 10 min. The final supernatants, designated as HAHp(3.0)/ZnO NPs, were collected, freeze dried, and stored at 4 °C for future experiments. The peptide concentration of dried powder of HAHp(3.0)/ZnO NPs was 9.23 g/kg, determined by *ortho*-phthaldialdehyde (OPA) method [73], with GSH as the standard. The total content of zinc in the HAHp(3.0)/ZnO NPs was 9127.4 mg/kg as determined by inductively coupled plasma-optical emission method [11].

### 4.3. Experimental Animals

Female and male Kunming species mice (clean), weighing approximately 18–22 g, were acclimatized in the animal facility for 4 days on standard mice chow and allowed water ad libitum under controlled conditions of temperature (20–24 °C), relative humidity (55–70%), and in a quiet room with 12/12 h light/dark cycle. Experimental procedures were performed in accordance with the Guide for the Care and Use of Laboratory Animals (issued by the Ministry of Science and Technology of the People’s Republic of China in 2006). The research was conducted in accordance with the Guide for the Care and Use of Laboratory Animals (National Research Council, 2010). The animal experiments were approved by the Ethics Committee of Experimental Animal Care at Zhejiang Ocean University.

### 4.4. Acute Oral Toxicity Study

The acute oral toxicity of HAHp(3.0)/ZnO NPs was evaluated in mice using the limit test method according to the procedures outlined in the Procedure and Methods of Food Safety Toxicological Assessment, GB15193.3-2014 (in Chinese) (National Health and Family Planning Commission of the People’s Republic of China, 2015). After acclimatization, the mice were randomly assigned to two groups. Each group contained 20 animals (10 female and 10 male). The BW of each mouse was measured prior to dosing on the first day. HAHp(3.0)/ZnO NPs conjugates were dissolved in saline water to reach the concentration of 1.0 g/mL and then administered by oral gavage at a dose of 10.0 g/kg BW. The CK mice were orally administered sterile saline (0.2 mL/kg BW). The clinical signs were monitored continuously for the first 1 h after administration. The general condition, toxic symptoms, and mortality were monitored daily for 14 days after administration. On the 15th day, all of the mice were weighed and killed under light ether anesthesia. The liver, thymus, and spleen tissues were removed and weighed. The liver coefficient, thymus coefficient, and spleen coefficient of the CK and HAHp(3.0)/ZnO NPs groups were calculated according to the following equations:

Liver coefficient/(g/kg) = Liver weight (g)/(Mouse weight (g) × 1000)(1)

Thymus coefficient/(mg/10 g) = Thymus weight (mg)/(Mouse weight (g) × 1)(2)

Spleen coefficient/(mg/10 g) = Spleen weight (mg)/(Mouse weight (g) × 1)(3)

### 4.5. Continuous Oral Administration of HAHp(3.0)/ZnO NPs in Mice

After getting acclimatized to the animal facility for 4 days on standard mice chow and having access to water under controlled conditions, mice were divided into two groups of 10 animals each (5 female and 5 male). Animals in the CK group were orally administered 0.2 mL of saline daily. The treated group was given a dose of 1.0 g/kg BW HAHp(3.0)/ZnO NPs daily for 14 days. The fecal samples from the first day (0 day) and the seventh day (7 day) were collected, mixed separately based on sex, and stored at −80 °C for further use. After completing the oral administration on the 14th day (14 day), the mice were not permitted to eat chow, but they were allowed to freely drink water. Animals were killed at 4 h after the last oral administration under light ether anesthesia. The small intestines were removed and processed for histopathology. The fecal samples at the time of the killing were collected aseptically from the descending colon directly, mixed together according to sex, and stored at −80 °C until DNA isolation. The liver tissues were collected after absorbing excess blood with blotting paper. Some of liver sections were blended with ice-cold saline at a ratio of 1:9 (*w*/*v*) and homogenized in an ice bath to prepare 10% liver homogenates, followed by centrifugation at 2500× *g* for 10 min at 4 °C. The supernatants were collected and stored at −80 °C for further oxidative status assay within 1 week.

### 4.6. Histopathology

The histopathology of jejunum was assessed via HE stain following a standard procedure. Briefly, paraffin sections (5 μm-thick) were cut, de-paraffinized in xylene, rehydrated in an alcohol gradient, and stained with HE. Stained sections were observed using a light microscope (CX41-12C02, Olympus, Tokyo, Japan) at 200× magnification.

### 4.7. DNA Isolation and Polymerase Chain Reaction (PCR) Amplification and Sequencing

The DNA of the total bacteria in the fecal samples separated based on sex at 0 day, 7 day, and 14 day were extracted according to the DNA extraction kit instructions. All of the operations strictly followed the product specifications. The total bacteria DNA of the fecal samples was used as the template for PCR amplification of the bacterial 16S rRNA. All extracted DNA were forwarded to Hangzhou Legenomics Bio-Pharm Technology Co., Ltd. (Hangzhou, China) for PCR analysis and high-throughput sequencing. The specific primers 338F (5′-ACTCCTACGGGAGGCAGCA-3′) and 806R (5′-GGACTACHVGGGTWTCTAAT-3′) with a barcode were used for amplification. TransStart FastPfu DNA Polymerase (TransGen AP221-02; 20 μL reaction system) was used for the PCR (Applied Biosystems^®^ GeneAmp^®^ 9700, Thermo Fisher Scientific Inc., Waltham, MA, USA). The 16S rRNA fragment V3–V4 variable region was the amplification substance of PCR. The amplification program was based on the following optimal annealing and extension conditions: 95 °C for 3 min, 25 cycles (95 °C for 30 s, 55 °C for 30 s, and 72 °C for 45 s), and 72 °C for 5 min, with a hold at 4 °C. This was repeated three times for each specimen. The PCR amplification products from the same specimen were mixed and subjected to 2% agarose gel electrophoresis. The target products were collected with gel extraction using the AxyPrep DNA Gel Extraction Kit (Axygen, Union City, CA, USA), eluted with Tris-HCl, and analyzed with 2% agarose gel electrophoresis. In order to ensure the accuracy and reliability of data analysis in the subsequent assay, PCR amplification needed to meet the following requirements: (1) used a low cycle number in amplification whenever possible; and (2) ensured that the number of cycles amplified per sample was consistent. Moreover, a representative sample was selected at random to perform preliminary experiments, and the majority of the samples could be amplified at the lowest number of cycles to produce a suitable concentration. The quantitative detection of PCR amplification products was further performed using a QuantiFluor™-ST blue fluorescence quantitative system (Promega, Madison, WI, USA). After the construction of a MiSeq library, the MiSeq high-throughput sequencing procedures were performed. The paired-end (PE) reads obtained in MiSeq sequencing were first mosaicked according to the overlap relationships. After removing the low-quality sequences, the relevant raw data were analyzed with procedures that identified and clustered the 16S rRNA sequences. The number of operational taxonomic units (OTUs, with a similarity of 97%) was calculated for each specimen.

### 4.8. Bioinformatics Analysis

Rarefaction curves of the OTUs were drawn based on the sequencing results, using a specific taxon to represent a particular species. Alpha-diversity was calculated on the Chao1 index (a community richness index) and the Shannon index (a community diversity index). Beta diversity (between communities) was applied using PCoA for the statistical analysis of the OTUs data. The species abundance at the phylum and genus levels was determined. Significant differences between the CK and HAHp(3.0)/ZnO NPs administration groups were compared at various taxonomic levels. The 16S rDNA sequencing data of intestinal microbiota was further analyzed by an online tool PICRUSt (http://picrust.github.io/picrust/tutorials/algorithm_description.html). Potential microbial functions were identified using eggnog functional prediction, and the 16S rRNA sequences were compared with Greengenes database to obtain COG orthology and function abundance. The relative abundance of each function was then calculated based on the total abundance.

### 4.9. Determination of SCFAs in Feces

Fecal samples (20 mg of freeze dried powder) were mixed with 1.2 mL of 0.1 mol/L phosphate buffered saline (PBS) (pH 6.5). The mixture was vortexed for 2 min and centrifuged at 5000× *g* for 5 min. The supernatant (1 mL) was collected, blended with 2.4 mL of absolute ethanol, and vortexed completely. Then, 850 μL of solution was removed to an ampere bottle, followed by addition of 1 mL of n-hexane and 100 μL of concentrated sulfuric acid. After sealed with alcohol burner, the ampere bottle was kept at room temperature for 1 h, and incubated at 60 °C water bath for 1 h. The upper layer of solutions was finally filtered through a 0.22 μm organic filter into HPLC vials.

The analysis of SCFAs in feces was performed according to the method described by Asarat, Vasiljevic, Ravikumar, Apostolopoulos and Donkor (2016) with slight modifications [74]. Briefly, the HPLC system used was an Agilent 1260 Infinity (Waldbronn, Germany) equipped with a quaternary pump, an autosampler, a column oven and a variable wavelength UV-vis detector (PDA). A Zorbax SB-C_18_ column (4.6 × 250 mm, 5 μm) was used at 30 °C for HPLC analysis. Sample solution (10 μL) was injected into the HPLC system and eluted with a mobile phase made of a mixture of 50 mM PBS (pH 2.8) and acetonitrile at a ratio of 99:1 (*v*/*v*). The flow rate was 1.0 mL/min running in isocratic conditions for 35 min and the wavelength was set at 210 nm. A mixture of four standards of SCFAs including formic, acetic, propionic and butyric acids, with concentrations ranging from 10,000 to 5 mg/L, was performed at the same conditions. The concentrations of formic, acetic, propionic and butyric acids in fecal solution were calculated according to their corresponding standard curves.

### 4.10. Determination of Amino Acid in Feces

The amino acid composition of fecal samples was determined according to our previous method [75]. In this study, the concentration of amino acid was expressed as μmol per L.

### 4.11. Determination of Oxidative Status

After continuous oral administration of HAHp(3.0)/ZnO NPs for 14 days, the activities of some key enzymes in the liver, including SOD, GPx, and CAT, were measured using their corresponding assay kits. The content of antioxidant compounds and the oxidative degree of lipids are also associated with the oxidative stress. In the present study, the concentration of GSH, one of major antioxidants in organisms, as well as MDA, which reflects the finial oxidation degree of lipids, were also determined. The supernatants of 10% liver homogenates were used for GSH and MDA assays. The 1% and 0.25% liver homogenates were prepared by diluting the supernatant of 10% liver homogenates with saline. The 1% liver homogenates were used for the CAT assay, and the 0.25% liver homogenates were used for the total SOD and GPx assays.

Meanwhile, the concentrations of protein, peptide and total SH were compared between HAHp(3.0) and HAHp(3.0)/ZnO NPs solutions. The protein concentration was determined using Bradford’s (1976) method with bovine serum albumin as the standard [76]. The peptide concentration was measured using OPA method [73]. Both protein and peptide concentrations were expressed as mg per mL. The total SH concentration was evaluated using the total SH assay kit and expressed as mmol per gprot.

### 4.12. Statistical Analysis

The data were expressed as mean ± standard deviation. Statistical analysis was performed using the SPSS^®^ software (SPSS Statistical Software 19.0, Inc., Chicago, IL, USA). The significant difference was set as *p* < 0.05.

## Figures and Tables

**Table 1:** Comparing body weight and coefficients of the liver, thymus, and spleen between the normal control and HAHp(3.0)/ZnO NPs treatment groups in the acute toxicity experiment.

Group	Body Weight/g		Liver Coefficient/(g/kg)	Thymus Coefficient/(mg/10 g)	Spleen Coefficient/(mg/10 g)
	0 Day	14 Day			
**CK**	F: 19.79 ± 1.93 ^a^	21.10 ± 1.32 ^a^	F: 46.41 ± 3.70 ^a^	F: 33.34 ± 5.85 ^a^	F: 40.49 ± 5.57 ^b^
	M: 22.13 ± 1.35 ^b^	23.93 ± 1.82 ^b^	M: 48.33 ± 3.14 ^a^	M: 26.01 ± 1.14 ^a^	M: 30.44 ± 6.77 ^a^
**HAHp(3.0)/ZnO NPs**	F: 19.86 ± 0.83 ^a^	20.87 ± 1.24 ^a^	F: 45.29 ± 5.56 ^a^	F: 31.67 ± 7.64 ^a^	F: 40.32 ± 6.38 ^b^
	M: 21.83 ± 1.59 ^b^	22.54 ± 2.31 ^ab^	M: 48.29 ± 3.42 ^a^	M: 25.50 ± 9.27 ^a^	M: 30.28 ± 2.39 ^a^

CK, normal control. Each group contained 10 female (F) and 10 male (M) mice. Different superscript letters in the same column indicate significant differences between two samples (*p* < 0.05).

**Table 2:** Comparing relative abundance of bacteria community in fecal samples at the genus level between 14 d CK(F) and 14 d Z(F).

Genus	Taxon	14 d CK(F)	14 d Z(F)	*p*
*Bacteroidales_S24-7_group_norank*	p__Bacteroidetes; c__Bacteroidia; o__Bacteroidales; f__Bacteroidales_S24-7_group; g__norank	23.41	12.40	*
*Lactobacillus*	p__Firmicutes; c__Bacilli; o__Lactobacillales; f__Lactobacillaceae; g__Lactobacillus; s__Lactobacillus_murinus	11.34	15.51	*
*Rikenellaceae_RC9_gut_group*	p__Bacteroidetes; c__Bacteroidia; o__Bacteroidales; f__Rikenellaceae; g__Rikenellaceae_RC9_gut_group; s__unidentified	6.07	0.94	*
*Alistipes*	p__Bacteroidetes; c__Bacteroidia; o__Bacteroidales; f__Rikenellaceae; g__Alistipes; s__uncultured_bacterium	3.54	0.75	*
*Desulfovibrio*	p__Proteobacteria; c__Deltaproteobacteria; o__Desulfovibrionales; f__Desulfovibrionaceae; g__Desulfovibrio; s__uncultured_bacterium	3.29	3.02	-
*Helicobacter*	p__Proteobacteria; c__Epsilonproteobacteria; o__Campylobacterales; f__Helicobacteraceae; g__Helicobacter; s__Helicobacter_ganmani	2.94	0.87	-
*Odoribacter*	p__Bacteroidetes; c__Bacteroidia; o__Bacteroidales; f__Porphyromonadaceae; g__Odoribacter; s__unidentified	3.02	0.86	*
*Erysipelotrichaceae_unclassified*	p__Firmicutes; c__Erysipelotrichia; o__Erysipelotrichales; f__Erysipelotrichaceae	3.00	1.68	*
*Bacteroides*	p__Bacteroidetes; c__Bacteroidia; o__Bacteroidales; f__Bacteroidaceae; g__Bacteroides	4.11	0.31	*
*Acetitomaculum*	p__Firmicutes; c__Clostridia; o__Clostridiales; f__Lachnospiraceae; g__Acetitomaculum; s__uncultured_bacterium	1.81	0.51	-
*Lachnoclostridium*	p__Firmicutes; c__Clostridia; o__Clostridiales; f__Lachnospiraceae; g__Lachnoclostridium	2.19	2.85	-
*Ruminococcaceae_UCG-014*	p__Firmicutes; c__Clostridia; o__Clostridiales; f__Ruminococcaceae; g__Ruminococcaceae_UCG-014; s__unidentified	1.71	7.26	*
*Enterorhabdus*	p__Actinobacteria; c__Actinobacteria; o__Coriobacteriales; f__Coriobacteriaceae; g__Enterorhabdus; s__uncultured_bacterium	1.67	2.03	-
*[Eubacterium]_coprostanoligenes_group*	p__Firmicutes; c__Clostridia; o__Clostridiales; f__Ruminococcaceae; g__[Eubacterium]_coprostanoligenes_group	1.58	0.45	-
*Candidatus_Saccharimonas*	p__Saccharibacteria; c__Unknown_Class; o__Unknown_Order; f__Unknown_Family; g__Candidatus_Saccharimonas; s__uncultured_bacterium	1.70	2.69	*
*Lachnospiraceae_uncultured*	p__Firmicutes; c__Clostridia; o__Clostridiales; f__Lachnospiraceae; g__Lachnoclostridium	1.34	1.15	-
*Lachnospiraceae_unclassified*	p__Firmicutes; c__Clostridia; o__Clostridiales; f__Lachnospiraceae; g__Lachnoclostridium	1.19	3.62	-
*Rikenella*	p__Bacteroidetes; c__Bacteroidia; o__Bacteroidales; f__Rikenellaceae; g__Rikenella; s__Rikenella_microfusus_DSM_15922	1.28	0.38	-
*Streptococcus*	p__Firmicutes; c__Bacilli; o__Lactobacillales; f__Streptococcaceae; g__Streptococcus; s__Streptococcus_acidominimus	1.04	0.30	*
*Roseburia*	p__Firmicutes; c__Clostridia; o__Clostridiales; f__Lachnospiraceae; g__Roseburia	1.54	2.40	-
*Ruminococcaceae_UCG-013*	p__Firmicutes; c__Clostridia; o__Clostridiales; f__Ruminococcaceae; g__Ruminococcaceae_UCG-013	1.01	2.44	*
*Coriobacteriaceae_unclassified*	p__Actinobacteria; c__Actinobacteria; o__Coriobacteriales; f__Coriobacteriaceae	0.88	0.73	-
*Lachnospiraceae_UCG-006*	p__Firmicutes; c__Clostridia; o__Clostridiales; f__Lachnospiraceae; g__Lachnospiraceae_UCG-006; s__uncultured_bacterium	0.81	1.52	-
*[Eubacterium]_xylanophilum_group*	p__Firmicutes; c__Clostridia; o__Clostridiales; f__Lachnospiraceae; g__[Eubacterium]_xylanophilum_group; s__uncultured_bacterium	0.85	1.43	-
*Ruminococcaceae_UCG-010*	p__Firmicutes; c__Clostridia; o__Clostridiales; f__Ruminococcaceae; g__Ruminococcaceae_UCG-010; s__unidentified	0.84	0.69	-
*Aerococcus*	p__Firmicutes; c__Bacilli; o__Lactobacillales; f__Aerococcaceae; g__Aerococcus	1.07	3.29	*
*Mollicutes_RF9_norank*	p__Tenericutes; c__Mollicutes; o__Mollicutes_RF9; f__norank; g__norank	0.47	0.69	-
*Lachnospiraceae_NK4A136_group*	p__Firmicutes; c__Clostridia; o__Clostridiales; f__Lachnospiraceae; g__Lachnospiraceae_NK4A136_group	0.47	1.54	-
*Ruminococcus_1*	p__Firmicutes; c__Clostridia; o__Clostridiales; f__Ruminococcaceae; g__Ruminococcus_1; s__unidentified	0.45	1.54	-
*Staphylococcus*	p__Firmicutes; c__Bacilli; o__Bacillales; f__Staphylococcaceae; g__Staphylococcus	0.43	1.20	*
*Vibrio*	p__Proteobacteria; c__Gammaproteobacteria; o__Vibrionales; f__Vibrionaceae; g__Vibrio	0.22	2.02	*
*Ruminococcaceae_unclassified*	p__Firmicutes; c__Clostridia; o__Clostridiales; f__Ruminococcaceae	0.19	0.96	*
*Ruminiclostridium_5*	p__Firmicutes; c__Clostridia; o__Clostridiales; f__Ruminococcaceae; g__Ruminiclostridium_5	0.16	0.89	*
*Marvinbryantia*	p__Firmicutes; c__Clostridia; o__Clostridiales; f__Lachnospiraceae; g__Marvinbryantia	0.15	1.45	*
*Lachnospiraceae_UCG-001*	p__Firmicutes; c__Clostridia; o__Clostridiales; f__Lachnospiraceae; g__Lachnospiraceae_UCG-001; s__unidentified	0.13	0.62	-
*Faecalibaculum*	p__Firmicutes; c__Erysipelotrichia; o__Erysipelotrichales; f__Erysipelotrichaceae; g__Faecalibaculum; s__uncultured_bacterium	0.13	5.95	*
*Coprococcus_3*	p__Firmicutes; c__Clostridia; o__Clostridiales; f__Lachnospiraceae; g__Coprococcus_3	0.07	0.87	*
*Bifidobacterium*	p__Actinobacteria; c__Actinobacteria; o__Bifidobacteriales; f__Bifidobacteriaceae; g__Bifidobacterium	0.01	0.53	*

Letters p, c, o, f, g, and s in Taxon represent phylum, class, order, family, genus and species, respectively. An asterisk represents significant differences between 14 d CK(F) and 14 d Z(F) in relative abundances (*p* < 0.05).
